# Effect of Resistant Dextrin on Intestinal Gas Homeostasis and Microbiota

**DOI:** 10.3390/nu14214611

**Published:** 2022-11-02

**Authors:** Claudia Barber, Carlos Sabater, María Ángeles Ávila-Gálvez, Fernando Vallejo, Rogger Alvaro Bendezu, Laetitia Guérin-Deremaux, Francisco Guarner, Juan Carlos Espín, Abelardo Margolles, Fernando Azpiroz

**Affiliations:** 1 Digestive System Research Unit, University Hospital Vall d’Hebron, 08035 Barcelona, Spain; 2 Departament de Medicina, Universitat Autònoma de Barcelona, 08193 Bellaterra, Spain; 3 Centro de Investigación Biomédica en Red de Enfermedades Hepáticas y Digestivas (Ciberehd), 28029 Madrid, Spain; 4 Department of Microbiology and Biochemistry, IPLA-CSIC, 33300 Asturias, Spain; 5 Health Research Institute of Asturias, ISPA, 33011 Asturias, Spain; 6 Laboratory of Food & Health, Group of Quality, Safety, and Bioactivity of Plant Foods, CEBAS-CSIC, 30100 Murcia, Spain; 7 Metabolomics Service, CEBAS-CSIC, Campus de Espinardo, 30100 Murcia, Spain; 8 Hospital General de Cataluña, Sant Cugat del Vallés, 08003 Barcelona, Spain; 9 Roquette Frères, 62136 Lestrem, France

**Keywords:** prebiotic, gut microbiota, metagenomics, metabolomics, digestive sensations, intestinal gas, resistant dextrin

## Abstract

Previous studies have shown that a resistant dextrin soluble fibre has prebiotic properties with related health benefits on blood glucose management and satiety. Our aim was to demonstrate the effects of continuous administration of resistant dextrin on intestinal gas production, digestive sensations, and gut microbiota metabolism and composition. Healthy subjects (*n* = 20) were given resistant dextrin (14 g/d NUTRIOSE^®^, Roquette Frères, Lestrem, France) for four weeks. Outcomes were measured before, at the beginning, end, and two weeks after administration: anal evacuations of gas during daytime; digestive perception, girth, and gas production in response to a standard meal; sensory and digestive responses to a comfort meal; volume of colonic biomass by magnetic resonance; taxonomy and metabolic functions of fecal microbiota by shotgun sequencing; metabolomics in urine. Dextrin administration produced an initial increase in intestinal gas production and gas-related sensations, followed by a subsequent decrease, which magnified after discontinuation. Dextrin enlarged the volume of colonic biomass, inducing changes in microbial metabolism and composition with an increase in short chain fatty acids-producing species and modulation of bile acids and biotin metabolism. These data indicate that consumption of a soluble fibre induces an adaptative response of gut microbiota towards fermentative pathways with lower gas production.

## 1. Introduction

Abnormal gas evacuation associated to gas-related symptoms, such as borborygmi, abdominal bloating, distension, and pain, are a frequent complaint in clinical practice. However, the relation of intestinal gas to symptoms is not well understood. Intestinal gas is produced by-and-large by the microbiota fermentation of meal components that escape small bowel absorption and reach the colon [1]. Hence, two factors determine intestinal gas metabolism: the substrates available from the diet and microbiota metabolic activity [2,3]. 

Prebiotics have been defined as substrates selectively utilized by host microorganisms that confer a health benefit [4]. However, microbiota metabolism of fermentable substrates may release gas, which may induce symptoms in susceptible individuals with increased sensitivity of the gut, such as patients with functional digestive disorders [1,2]. Studies in vitro have shown that a resistant dextrin soluble fibre is selectively consumed by Clostridium cluster XIVa and Roseburia genus and beneficially modifies microbiota metabolism [5]. Clinical studies confirmed the positive modulation of gut microbiota [6,7], and further showed that it is well-tolerated and exerts health benefits related to gut health, sustained energy, blood glucose management and satiety [8,9,10]. However, the effect of resistant dextrin on intestinal gas metabolism, a potential mechanism of digestive complaints, has not been investigated. We hypothesized that administration of resistant dextrin, induces an adaptation in gut microbiota metabolic activity and composition, and modulates the homeostasis of intestinal gas. Our objective was to identify the effects of continuous administration of resistant dextrin on intestinal gas production, digestive sensations, and gut microbiota metabolism and composition. Hence, we designed a pilot study to measure the effects of dextrin at initial administration, late administration, and post administration.

## 2. Materials and Methods

### 2.1. Study Design and Ethical Aspects

**Figure 1:**
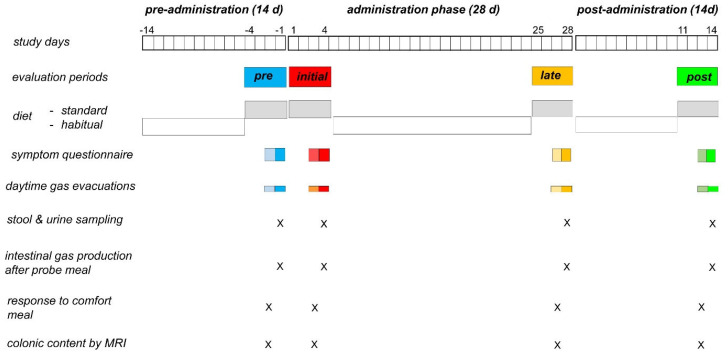
Experimental design and procedures. Open label, single arm, proof-of-concept study on the effect of a prebiotic fibre in healthy men. The study consisted of a pre-administration phase (14 d), an administration phase (28 d) and a post-administration phase (14 d). During 4-day evaluation periods at the end of the pre-administration phase (blue), early administration phase (red), late administration phase (orange) and end of the post-administration phase (green) the diet was standardized and the study outcomes were measured.

A proof-of-concept study testing the effect of a prebiotic fibre in healthy subjects in an open-label, single-arm design was performed in a tertiary referral center. The study consisted of a pre-administration phase (2 wk), an administration phase (4 wk) and a post-administration phase (2 wk). During 4-day evaluation periods (a) before administration, (b) initial administration phase, (c) late administration phase, and (d) end of the post-administration phase, the diet was standardized, and the study outcomes were measured (Figure 1). A commercial prebiotic fibre was used for its known health benefits proven by previous studies [8,9,10]. The study plan, hypothesis, and aims were independently drafted by FA, and discussed to reach a consensus with the rest of investigators. The study protocol was revised by the Institutional Review Board of the University Hospital Vall d’Hebron, (Comitè d’Ètica d’Investigació Clinica, Vall d’Hebron Institut de Recerca; protocol PR-AG 420-2018 approved 30 November 2018) and registered with ClinicalTrials.gov (NCT04164914). The research was conducted according to the Declaration of Helsinki. The participants were informed and signed a consent form to participate in the study. The execution of the experiments was performed by CB under the direct supervision of FA. Specific analyses were independently performed by the physiology (FA), metabolomic (JCE), and microbiota laboratories (AM), respectively, and later jointly interpreted by all investigators. This work is part of the doctoral thesis of CB directed by FA, Universitat Autònoma de Barcelona.

### 2.2. Participants

Healthy male individuals (*n* = 20) were recruited. Candidates completed a questionnaire to confirm the absence of digestive symptoms (symptoms scoring above 2 on scales graded from 0 to 10 were exclusion criteria), and hence, disorders of the digestive function were excluded using established criteria (Rome IV). Previous studies showed the power of the questionnaire to segregate patients and healthy individuals [2,11,12,13,14,15]. Candidates having consumed antibiotics, probiotics, or prebiotics 2 months previously were not included in the study. 

### 2.3. Intervention 

A prebiotic resistant dextrin (14 g/d NUTRIOSE^®^ FB06 soluble fibre, Roquette Frères, Lestrem, France) was administered in the morning (powder dissolved in water) during the 4 wk administration phase. This particular brand of resistant dextrin was chosen based on previous studies showing its modulatory effects on intestinal microbiota [5,6,7], as well as potential health benefits [8,9,10]. 

### 2.4. Diet

During the evaluation periods (4 days pre-administration, initial administration, late administration, and end of the post-administration phase), participants were put on a diet low in fibre (estimated to provide 7 g of fibre per day) complemented with one portion per day (adjusted to contain 12 g of fibre) of a specific foodstuff, so that the total fibre load of the standard diet was 19 g per day. The diet low in fibre was restricted to: (a) fowl, meat, eggs and fish; (b) bread, pasta and rice; (c) green salad; (d) apples, pears, berries, tangerine and strained orange juice; and (e) dairy products [11]. The fibre-containing complements were Brussels sprouts, artichoke, peas, chickpeas, beans, lentils, whole crackers, prunes, peach, or banana. During the rest of the study (out of the evaluation periods) participants were instructed to follow their habitual diet. During the entire study period, participants were instructed to avoid any product containing prebiotics, probiotics, and fermented dairy products (Figure 1). Adherence to the diet was reinforced at each visit.

### 2.5. Outcomes

#### 2.5.1. Evacuations of Gas Per Anus (Primary Outcome) 

Participants were instructed to measure the number anal gas evacuations during daytime the last 2 days of the periods of evaluation by means of a hand tally counter (No 101, Digi Sport Instruments, Shanggiu, China) (Figure 1). The results obtained by this technique have been shown to be consistent and reproducible [2,11]. Furthermore, data registered by the event marker closely correlate with quantitative measurements of the volume of gas evacuated per anus [16,17,18,19]. 

#### 2.5.2. Daily Symptom Questionnaire

Parameters related to digestive function were collected by questionnaires to be filled at the end of the last 2 days of the periods of evaluation. (Figure 1). Abdominal sensations were measured using analogue scales, as follows; scales graded from 0 (not at all) to 10 (very intense) were used to measure: (a) flatulence (sensation of gas evacuation per anus), (b) discomfort/pain, (c) distension (subjective sensation of increase in girth), (d) borborygmi (abdominal rumbling), and (e) bloating (sensation of fullness or pressure); scales graded from +5 (very positive sensation) to −5 (very negative sensation) were used to measure well-being (sensation of digestive pleasant/unpleasant sensation) and mood. The questionnaires also included items on bowel habit (number of evacuations, and stool consistency evaluated by the Bristol scale), and weight of stools measured by a digital balance (BT- 32013, El Corte Ingles, Madrid, Spain). Previous studies showed the sensitivity of this questionnaire to detect differences in the responses to dietary interventions under various conditions [2,11,15,20]. 

#### 2.5.3. Effects Induced by a Pleasant Meal 

On the third day of the periods of evaluation at the end of the pre-administration phase, early administration phase and late administration phase, a comfort meal (warm ham and cheese sandwich plus orange juice containing 425 Kcal with 47 g carbohydrates, 18 g proteins 17 g fat) was administered (Figure 1). Digestive sensations (hunger/satiety, abdominal fullness, discomfort/pain, desire of choice eating, digestive well-being, and mood) were measured using graded scales before ingestion, and at 0 min, 30 min and 60 min after ingestion. Emptying of the stomach and gallbladder was measured using an ultrasonographic technique (Supplementary Material).

#### 2.5.4. Intestinal Gas Production 

On the fourth day of the periods of evaluation at the end of the pre-administration phase, early administration phase and late administration phase a gas production test was performed (Figure 1). After fasting overnight, participants reported to the research unit and were instructed to eat a probe meal. The probe meal consisted of 96 g oatmeal cookies (Digestive Avena, Fontaneda, Madrid, Spain) and 200 mL coffee with milk (540 Kcal; 63.5 g carbohydrates, 24.2 g lipids, 13.2 g proteins, 8.4 g fibre). During 4 h in the postprandial period, the gas volume collected via a rectal tube (20 F Foley catheter, Bard, Barcelona, Spain) was continuously measured using a computerized barostat, as previously described [2,21,22]. Changes in girth at the level of the umbilicus were measured at the beginning and at the end of the test using a sliding belt with 1 mm marks [23]. The sensitivity and reproducibility of this technique have been previously shown [13,24,25,26,27]. 

#### 2.5.5. Colonic Content Measurement

On the fourth day of the periods of evaluation at the end of the pre-administration phase, early administration phase and late administration phase, the volume of content within the colon was measured (Figure 1). Magnetic resonance imaging (MRI) of the abdomen was performed (1.5-T; Aera, Siemens Healthcare, Erlangen, Germany) without administration of contrast, and original software was used to measure the volume of colonic content, as previously described [28].

#### 2.5.6. Microbiota Composition and Functionality

On the third day of the periods of evaluation at the end of the pre-administration phase, early administration phase, late administration phase and post-administration phase, participants were instructed to collect faecal samples, which were immediately homogenized and frozen at −20 °C (Figure 1). Using a freezer pack, the samples were transported to the research unit, and were then stored for later analysis at −80 °C. Total DNA extracted was submitted to an external BaseClear sequencing service. Paired-end sequence reads (2 × 150 bp) showing an average minimum of 25 million reads per sample were generated using the Illumina NovaSeq system under accreditation according to the scope of BaseClear B.V. Sequencing experiments yielded 7.5 G output/sample. Reads were demultiplexed to generate FASTQ read sequence files using bcl2fastq2 (v2.18). Initial quality assessment was based on data passing the Illumina Chastity filtering. Subsequently, reads containing PhiX control signal were removed using an in-house filtering protocol. In addition, reads containing (partial) adapters were clipped (up to a minimum read length of 50 bp). The second quality assessment was based on the remaining reads using the FASTQC quality control tool version (v0.11.9).

Contaminant reads and low-quality sequences were separated in silico from microbial reads using Kneaddata (v0.7.4) and Trimmomatic (v0.39) software. For this purpose, the minimum length of output reads was computed as 50 percent of the length of the input reads considering a sliding window of 4:20. Bowtie2 (v2.4.2) was used to map metagenomic reads [29] against the databases of reference “*Homo sapiens* hg37 and human contamination Bowtie2” (v0.1) in order to remove host contamination.

Functional and taxonomic analysis of microbial communities at the species level was performed using the latest version available of MetaPhlAn 3.0 (v3.0.4) and HUMAnN 3.0 (v3.0.0), respectively [30,31]. In this sense, the latest versions of reference databases were selected: ChocoPhlAn (version “mpa_v30_ChocoPhlAn _ 201901”) database containing clade-specific marker genes for taxonomic identification and UniRef90 (version “uniref90_201901”) protein database to determine the abundance of microbial gene families and metabolic pathways. The abundances of gene families and metabolic pathways were re-normalized and expressed in units of copies per million. Alpha and beta diversity estimators were calculated. Only those clades showing at least 0.1% abundance in 50% of samples were considered, to filter rare taxa. Similarly, only genes showing at least 1 million reads present in at least 50% samples were selected when performing functional analysis. An additional beta-diversity analysis of microbial communities was performed following the Bray–Curtis dissimilarity method [32] implemented in the Phyloseq R package [33]. Ordination plots describing the distribution of microbial communities across individuals were generated using the Microbiome R package [34]. 

Statistical differences in taxonomic clades abundances between samples taken at different intervention times, as well as microbial gene families and metabolic pathways expression, were calculated using MaAsLin2 [35]. This package was developed specifically for a multivariable association between phenotypes and microbial metaomic features. Species showing *p_adj_* values (corrected by Benjamini–Hochberg method) lower than 0.05 were considered to select only relevant differences. All statistical tests and models were performed on R (v4.1.1). 

The raw sequences data were deposited in the Sequence Read Archive (SRA) of the NCBI (https://www.ncbi.nlm.nih.gov/sra, accessed on 25 October 2022) under bioproject code PRJNA892265.

#### 2.5.7. Metabolomic Analysis

On the third day of the periods of evaluation at the end of the pre-administration phase, early administration phase, late administration phase and post-administration phase, morning urine samples were collected by the participants, and immediately transported to the research unit, where they were stored at −80 °C for later analysis (Figure 1). 

### 2.6. Reagents

L-valine, 5-aminovaleric acid, N,O-bis-(trimethylsilyl) trifluoroacetamide (BSTFA), trimethylchlorosilane (TMCS), tert-butyl methyl ether (MTBE), N-(5-methyl-3-oxohexyl)alanine, glucoheptonic acid, homovanillic acid, dimethoxy benzoic acid, pipecolic acid, diaminopelargonic acid, imidazol lactate, glyceric acid, rhamnose, D-xylose, 3-hydroxyvaleric acid, sorbitol, mannitol, arabitol, short-chain fatty acids (SCFAs; acetic, propionic, isobutyric, butyric, isovaleric, and valeric acids), ursodeoxycholic acid, chenodeoxycholic acid, deoxycholic acid, cholic acid, acetonitrile (ACN), formic acid, and methanol (MeOH) were purchased from Sigma-Aldrich (St. Louis, MO, USA). 

### 2.7. Untargeted Urine Metabolomics Analysis by UPLC-QTOF-MS

Enzymatically hydrolyzed and non-hydrolyzed urine samples were analyzed by UPLC-ESI-QTOF-MS, as previously reported [3]. As reported elsewhere, creatinine was measured to allow diuresis standardization [36]. The equipment consisted of an Agilent 1290 Infinity series LC system coupled to a 6550 I-Funnel Accurate-Mass QTOF (Agilent Technologies, Waldbrom, Germany) with a dual electrospray ionization interface (ESI-Jet Stream Technology, Waldbroon, Germany) for simultaneous spraying of a mass reference solution that enabled continuous calibration of detected *m/z* ratios (Supplementary Material). Feature extraction was carried out on the Agilent Profinder B.06.00 software, a stand-alone feature extraction program for LC-MS-based profiling analyses (Supplementary Material). Determining compounds by molecular features (MFs) was carried out using a pre-filter to take peaks with a height greater than or equal to 10,000 counts, allowing only -H and -HCOO as negative ions species and +H as positive ions (Supplementary Material). Bile acids were analyzed in urine samples (with and without enzymatic treatment) using the same LC system, with some modifications (Supplementary Material).

### 2.8. Urine and Fecal Metabolomics Analysis by GC-MS

Non-hydrolyzed urine samples (1 mL) were centrifuged (14,000× *g* for 10 min) and dried overnight at 25 °C with a speed vacuum. Then, the samples were dissolved in 30 µL of pyridine and converted to trimethylsilyl derivatives by adding 30 µL of BSTFA, containing 1% TMCS. The chemical reaction was performed by heating at 100 °C for 15 min. Then, 1 µL of this reaction mixture was injected into the gas chromatograph. Silylated samples were analyzed using an HP 8890 gas chromatograph interfaced with an HP 5977B mass selective detector (Agilent) (Supplementary Material). The data were processed and quantified with the Mass Hunter software from Agilent. Compound identification was performed by comparing with the chromatographic retention characteristics and mass spectra of available authentic standards, the reported mass spectra, and the mass spectral library of the GC–MS data system (NIST 2010). The sum of extracted ion chromatograms of the ions associated with a compound was used for quantification.

Short chain fatty acids (SCFAs) were extracted from fecal samples (100 mg) with 1 mL of MTBE, using vortex for 3 min, and centrifugation at 10,000 rpm and 4 °C for 10 min. Finally, 1 µL of the upper organic phase was injected into the gas chromatograph. The samples were analyzed using the same HP 8890 gas chromatograph and following the methodology previously described by [37]. The data were processed and quantified as described above. 

### 2.9. Ancillary Study 

Interventions testing 40 g or more of dietary fibres have shown changes in gut microbiome composition and functionality relevant for health outcomes, such as improving glycemic homeostasis in Type 2 diabetes or immune status in healthy volunteers. Tolerance is a potential drawback for this type of interventions [38,39]. To evaluate dose-related effects in the initial and late responses of the main outcome, 10 participants underwent a second study with the administration of resistant dextrin at triple dose (42 g/d NUTRIOSE^®^ soluble fibre) for 18 days. As in the main study, the diet was standardized during the 4-day evaluation periods pre-administration, initial administration, and late administration phase, while during the rest of the study participants consumed their habitual diet (see Section 2.4 above). Following the same procedures as in the main study, during the last 2 days of the periods of evaluation, anal gas evacuations (number during daytime) and perception of abdominal sensations were measured (see Section 2.5 above). 

### 2.10. Statistical Analysis

#### 2.10.1. Sample size calculation

Estimation of the sample size was based on previous data [40], indicating that 20 subjects would be required to detect changes in the primary outcome of the main study (number of gas evacuations per anus during daytime) with 90% power and a 5% significance threshold. 

#### 2.10.2. General Statistics

Mean values (±SE) of the parameters evaluated were calculated. The normality of the data distribution was evaluated using the Kolmogorov–Smirnov test. The Student’s *t*-test was used to compare parametric data normally distributed; otherwise, paired data were analyzed by the Wilcoxon signed-rank test, and unpaired data by the Mann–Whitney *U* test. Linear regression analysis was applied to evaluate association of parameters. 

#### 2.10.3. Metabolomic Data

For statistical analysis, the final files were exported into the Mass Profiler Professional (MPP) 2.0 software package (Agilent, Santa Clara, CA, USA). The Shapiro-Wilk test was used to assess data normality. Data are expressed as the mean ± standard deviation (SD). Statistical comparisons were performed between the three phases of the study (four evaluation points) by the two-tailed paired Student’s *t*-test or Wilcoxon signed-rank test in normal and non-normal distributed data, respectively, with Bonferroni Holm family-wise error rate multiple testing corrections and a fold-change cut-off of 2-fold. Bristol and BMI values were used as possible covariates. A list of compounds that significantly differed between groups was generated. Both supervised methods (partial Least-squares discriminant analysis; PLS-DA), suitable for classification, and unsupervised methods (principal component analysis (PCA), clustering), appropriate for pattern identification, were used to visualize statistical results, using the final list of statistically significant MFs. When available, correlations between the change of specific SCFAs and polyols pair (final vs. initial) were performed using the Spearman’s or Pearson’s correlation coefficient test. Unidentified compounds were aligned across the samples based on their tolerance retention times and mass spectral similarity. The MassHunter MSC (Molecular Structure Correlator) program correlated accurate mass MS/MS fragment ions for a compound of interest with one or more proposed molecular structures for that compound (Supplementary Material). Statistically significant differences were considered at *p* < 0.05.

## 3. Results

### 3.1. Demographic Data 

Twenty subjects (healthy men between 19–41 years and body mass index between 20–27 Kg/m^2^) were recruited, completed the study, and were analyzed. 

### 3.2. Evacuations of Gas Per Anus 

**Figure 2:**
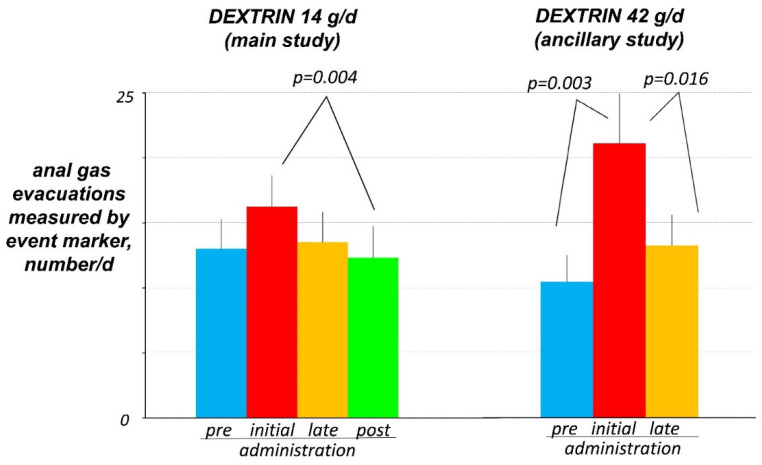
Number of daytime anal gas evacuations, during the last 2 days of the evaluation periods. Data are average of 2 consecutive days.

Before the administration phase, participants recorded a mean of 13 ± 2 evacuations of gas per day using the marker. At the beginning of the dextrin administration (14 g/d), the number of anal gas evacuations increased by 30 ± 13 % (initial phase), but the difference with pre-administration was not statistically significant (*p* = 0.126) (Figure 2). After four weeks of administration (late phase), the number of gas evacuations decreased back to the pre-administration level (*p* = 0.189 vs. initial phase), and two weeks after the end of administration (post-administration phase) the number of evacuations further reduced and became significantly smaller than at initial administration (*p* = 0.004 vs. initial phase).

The effect of dextrin on the number of evacuations of gas became clearer in the ancillary study when a higher dose of dextrin (42 g/d) was tested: the number of daytime anal gas evacuations significantly increased during the initial administration phase (by 172 ± 65 %; *p* = 0.001 vs. pre-administration); at the late administration phase (days 17 and 18) the number significantly declined back to the pre-administration level (*p* = 0.048 vs. initial administration; and *p* = 0.151 vs. pre-administration) (Figure 2). In the 10 subjects who underwent both tests, the initial increase and the later decay in the number of gas evacuations were higher with the high dose than with the low dose in the main study (*p* = 0.040 and *p* = 0.064, respectively). 

### 3.3. Digestive Perceptions and Bowel Habit

**Figure 3:**
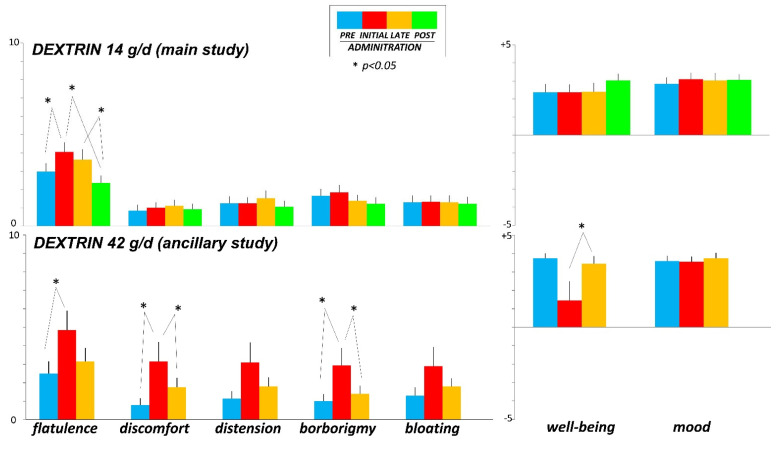
Digestive and hedonic sensations measured by daily questionnaires during the last 2 days of the evaluation periods. Data are average of 2 consecutive days.

Before dextrin administration, participants recorded (on the daily questionnaires) some degree of flatulence and borborygmi, associated with digestive well-being, positive mood and minimal scores of abdominal bloating, distension, and discomfort (Figure 3). Dextrin at initial administration increased the sensation of flatulence (by 35 ± 15%; *p* = 0.040 vs. pre-administration), but the sensation decreased during treatment and even further during the post-administration phase (*p* = 0.013 vs. early administration phase). A similar tendency was observed in the borborygmi scores, but without statistical significance. Dextrin administration did not induce abdominal symptoms and did not affect digestive well-being and positive mood reported by the participants during the pre-administration phase. Before administration, participants reported normal bowel habit, which was not affected by dextrin administration, except for a small, non-relevant reduction in stool consistency by the end of treatment (*p* = 0.049 vs. early phase), which reverted after administration (Supplementary Figure S1). 

The effect of dextrin on digestive sensations was magnified in the ancillary study testing a higher dose of dextrin (42 g/d) with an increase in the abdominal sensations and a drop in the sensation of digestive well-being at initial administration, which subsided by the end of the administration (Figure 2), and no changes in the normal bowel habit (Supplementary Figure S2). 

### 3.4. Response to the Comfort Meal

Dextrin administration did not disturb the normal responses to the comfort meal (homeostatic and hedonic sensations, gastric and gall bladder emptying) either at the initial or at the late administration phase (Supplementary Material).

### 3.5. Gas Production Test

**Figure 4:**
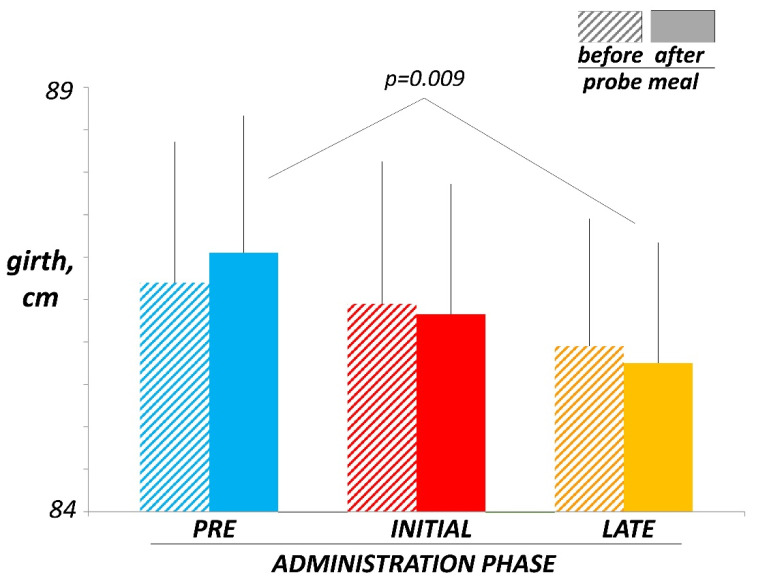
Effect of probe meal on girth. No significant changes were detected, but postprandial girth by the end of treatment (late administration) was smaller than pre-administration.

The meal was well tolerated by all participants without discomfort. In the pre-administration test, 205 ± 37 mL of gas were collected during the 4-h postprandial period. Dextrin administration did not induce significant changes in the gas volume collected after the meal, but a tendency towards an early increase (243 ± 41 mL during initial administration; *p* = 0.339 vs. pre-administration) and later decay during the administration period was observed (237 ± 49 mL during late administration; *p* = 0.919 vs. initial administration). Meal ingestion did not induce significant changes in girth in the pre-administration, initial, or late administration tests, but postprandial girth by the end of treatment was smaller than pre-administration (Figure 4).

### 3.6. Colonic Volume

Total colonic volume increased from 634 ± 52 mL before dextrin administration to 730 ± 57 mL at the beginning of administration (*p* < 0.001), and solid content increased from 494 ± 47 mL to 576 ± 52 mL (*p* < 0.001). The increase in volume persisted during the administration phase (726 ± 56 total volume and 552 ± 44 mL solid content by the end of administration; *p* = 0.007 and *p* = 0.035 vs. pre-administration, respectively). The increase in volume was larger in the proximal colon (solid content in ascending plus transverse colon was 286 ± 24 mL pre-administration vs. 345 ± 28 mL at initial and 342 ± 25 mL at late administration; *p* < 0.003 for both) than in the distal colon (solid content in descending plus pelvic colon was 205 ± 29 mL pre-administration vs. 231 ± 30 mL at initial and 210 ± 24 mL at late administration; *p* > 0.05 for both).

### 3.7. Metagenomic Study

**Figure 5:**
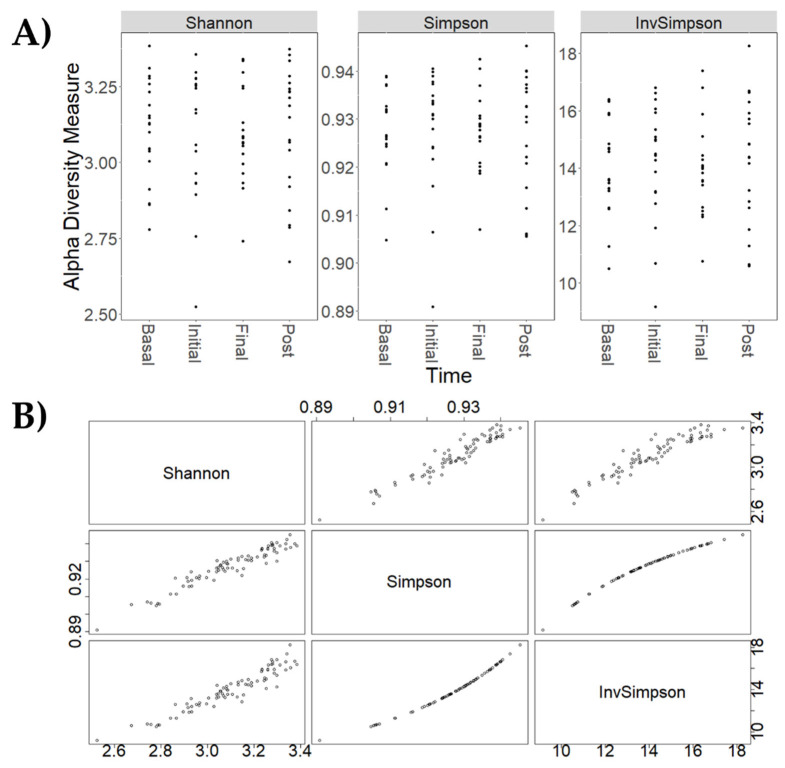
(**A**) Comparison of different alpha-diversity indicators (Shannon, Simpson and Inverse Simpson) of the relative abundance of taxa determined at different intervention periods: basal, initial intervention (initial), late intervention (final) and post-intervention (post). (**B**) Relationship between different alpha-diversity estimators: Shannon, Simpson and Inverse Simpson indices. These coefficients reflected similar patterns in the core microbiota.

**Figure 6:**
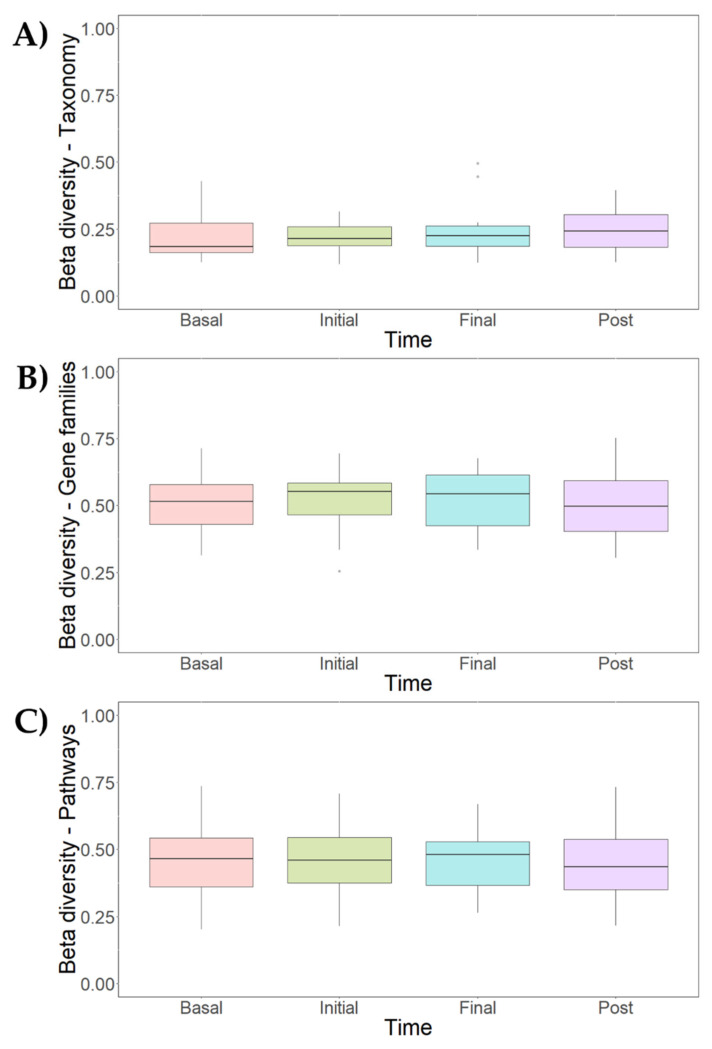
Beta-diversity analysis of taxonomic profiles (**A**), gene families (**B**) and metabolic pathways (**C**) found in the microbiota of participants at different intervention periods: basal, initial intervention (initial), late intervention (final) and post-intervention (post). Bray-Curtis method was selected for the calculation.

Changes in the metagenomic profiles of participants at different periods of the intervention (basal, initial, late, and post-administration) were determined at both taxonomic and functional level. Concerning taxonomic analysis, alpha diversity estimators measuring the variability of species within a sample were first calculated. The global Chao1 index, indicating of the number of species represented by only one individual in the sample, was 96.4 ± 6.8. Specific Chao1 indices for each intervention time were also calculated including basal (97.1 ± 6.5), initial (95.9 ± 7.5), late (95.9 ± 7.2) and post-intervention (96.9 ± 6.5) periods showing no major differences. Other alpha diversity estimators, such as Shannon, Simpson, and inverse Simpson indices, were calculated to characterize microbial diversity (Figure 5A). These coefficients reflected similar patterns in the core microbiota, confirming the results from the different analyses (Figure 5B). Although there were no relevant changes in microbial diversity within samples, a great dispersion was observed considering different intervention times. Finally, based on Bray–Curtis distances, beta diversity estimators were calculated (Figure 6). These parameters reflect differences in microbial diversity between samples. As can be seen, the number of microbial taxonomic clades slightly increased during the post-intervention period (Figure 6A). 

Diversity distances calculated by the Unifrac method were used to cluster metagenomes (Supplementary Figure S3A). Some samples corresponding to the same intervention period were grouped together although most metagenomes were grouped with samples from different periods. This may be attributed to the high variability between the microbiota of each individual, exerting a great influence on the global metagenomic profile, similar to the one exerted by the prebiotic supplementation itself. Nevertheless, according to dissimilarity indices, the gut microbiota composition was homogenous and no statistically significant changes (*p* < 0.05) assessed by the PERMANOVA method could be determined in the core microbiota depending on the intervention period. 

Regarding the functional analysis of metagenomes, gene count was determined for all intervention periods: basal (mean 88927; median 87225), initial (mean 84990; median 80854), final (mean 84366; median 86796) and post-intervention (mean 84620; median 77421). No statistically significant differences (*p* < 0.05) were found in the gene count of these groups. Beta-diversity was calculated using the Bray–Curtis method to measure differences in the expression of gene families and metabolic pathways (Figure 6B,C). No relevant differences were found in beta-diversity values of the global profiles of microbial gene families (Figure 6B) and metabolic routes expressed (Figure 6C). Moreover, metagenomic gene data were homogenous and no statistically significant differences (*p* < 0.05) in gene families and metabolic pathways according to the intervention time could be found by the PERMANOVA test. Similar to the taxonomic analysis, cluster analysis of functional profiles of metagenomes (Supplementary Figure S3B,C) highlighted the role of interindividual variability in sample distribution.

**Figure 7:**
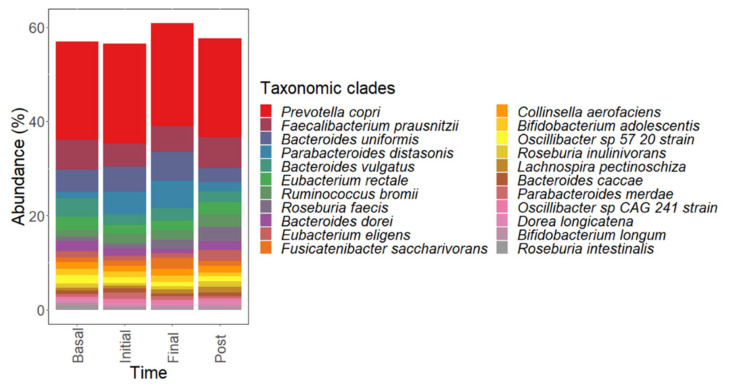
Graphical representation of the abundances of microbial species modulated by dextrin administration. Mean abundances of taxonomic species are illustrated at basal level, initial, late and post-intervention periods.

A principal coordinates analysis (PCoA) of complete microbial taxa present in the microbiota of each participant was computed to study further study individual differences in metagenomic profiles (Supplementary Figure S4). No characteristic patterns could be found in the global microbiota for different intervention periods when considering taxonomic (Supplementary Figure S4A) or functional profiles (Supplementary Figure S4B,C). This fact could be attributed to the high interindividual variability of the gut microbiota profiles. However, statistically significant changes in specific taxonomic profiles and metabolic functions after dextrin administration, were determined (Table 1 and Table 2). Concerning taxonomic clades stimulated by prebiotic administration (Table 1), an unclassified Firmicutes class and *Oscillibacter sp.* CAG 241 strain showed the highest abundances at initial dextrin intervention period. Similarly, *Tanerellaceae* family, *Parabacteroides,* and *Parabacteroides distasonis* showed the highest abundances at late dextrin intervention. Interestingly, a wide variety of taxa showed the highest abundances during the post-intervention period, including *Anaerostipes hadrus*, *Bifidobacterium longum*, *Blautia obeum*, *Dorea longicatena*, *Eubacterium eligens*, *Lachnospira pectinoschiza*, *Roseburia,* and *Ruminococcus* species. A graphical representation of major differences in the abundances of microbial species modulated in the intervention is provided in Figure 7. 

On the other hand, up to 8141 microbial gene families and 564 metabolic pathways were modulated by dextrin administration (Table 2). Most gene families showing the highest abundances at late intervention were found for *P. distasonis*, in agreement with taxonomic data (Table 1). Similarly, *R. bromii*, *R. torques, R. inulinovorans, B. obleum, R. faecis*, *E. eligens,* and *D. longicatena* exhibited many genes and metabolic pathways showing the highest abundances during the post-intervention period. In this sense, a broad range of metabolic functions were modulated by dextrin during initial and post-intervention periods, comprising ribonucleotide, amino acid, and carbohydrate metabolism.

### 3.8. Untargeted Urine Metabolomics

Multivariate analyses revealed that the concentration of 14 metabolites increased, and that of 12 metabolites decreased, measured in the positive ionization mode, after consuming dextrin, while 13 metabolites increased their concentration in the negative ionization mode after the administration phase (Supplementary Figure S5A). Of the total urine metabolites that significantly changed in the administration phase (*n* = 39), only a few of them yielded an accurate mass-based-putative identification when searched against several databases and online libraries. Finally, seven tentative urine metabolites were selected for further validation, considering a predictive score above 95% and a plausible biological implication in this dietary intervention, i.e., L-valine, N-(5-Methyl-3-oxohexyl) alanine, glucoheptonic acid, homovanillic acid, pipecolic acid, diaminopelargonic acid, and imidazole lactate. After validation with authentic standards and diuresis normalization, only the metabolite 7,8-diaminopelargonic acid was confirmed to be decreased by 1.6-fold after dextrin consumption. 

The number of metabolites that significantly changed after GC-MS analysis (at least 2-fold) in the three phases of the study was low. Only four metabolites significantly changed after the administration phase (Supplementary Figure S5B), although we searched for all the metabolites that significantly changed in the three phases and yielded an accurate mass-based-putative identification. Finally, seven metabolites were validated using authentic standards, i.e., glyceric acid and L-rhamnose (pre-administration phase), 3-hydroxyisovaleric acid (3-HVA), D-xylose, sorbitol and mannitol (administration phase), and arabitol (post-administration phase).

### 3.9. Targeted Analysis of Bile Acids and SCFAs

**Figure 8:**
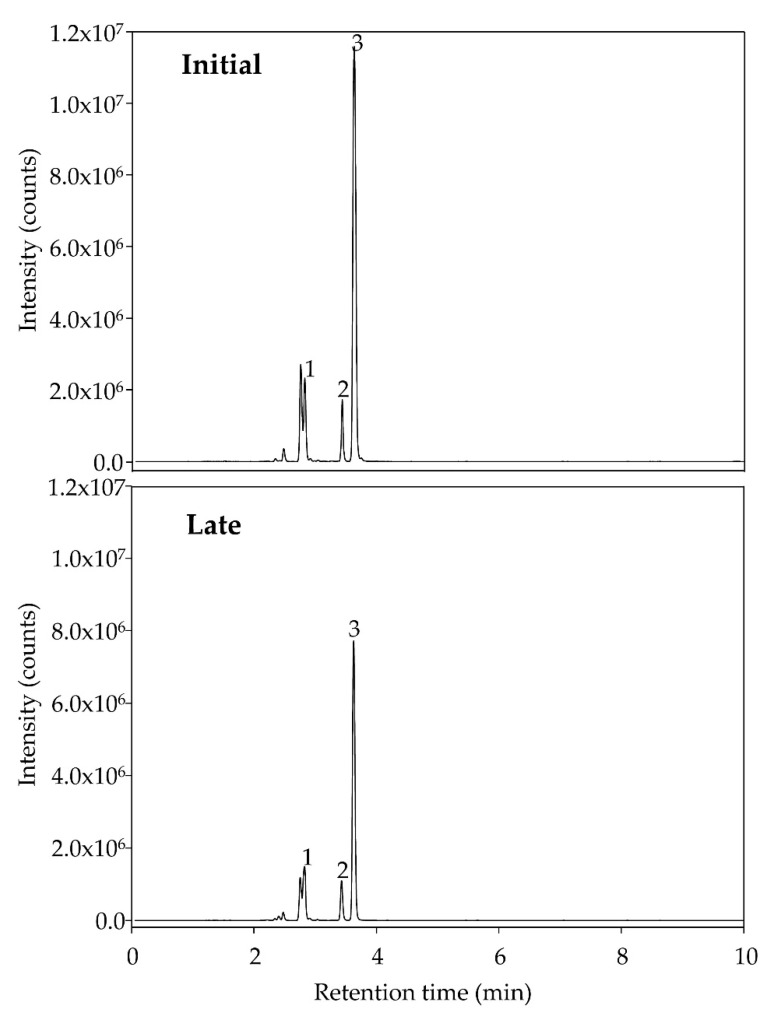
Extracted ion chromatograms of a volunteer showing a representative decrease in urine bile acids after consuming dextrin (late vs. initial); (1) deoxycholic acid, (2) ursodeoxycholic acid, and (3) chenodeoxycholic acid.

Since glucuronidated bile acids were not commercially available, their validation was achieved after detecting the corresponding free forms (commercially available) upon glucuronidase/sulfatase treatment. The primary bile acid cholate glucuronide increased 2.9-fold (*p* = 0.003) in the pre-administration phase. In contrast, the primary bile acid chenodeoxycholate glucuronide decreased 1.4-fold (*p* = 0.01), and the secondary bile acids, ursodeoxycholate glucuronide and deoxycholate glucuronide, decreased 1.4- and 2.2-fold (*p* = 0.04 and *p* = 0.02), respectively, upon dextrin consumption (Figure 8). 

**Figure 9:**
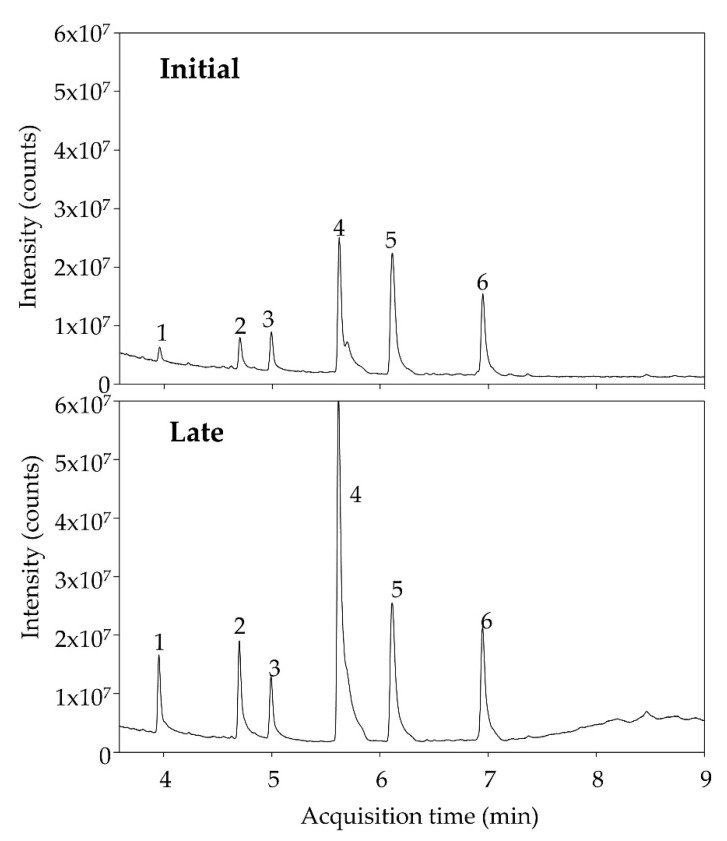
Representative chromatograms of fecal SCFAs before (Initial) and after (Late) consuming dextrin for 4 weeks. (1) Acetate; (2) Propionate; (3) Isobutyrate; (4) Butyrate; (5) Isovalerate; (6) Valerate.

Fecal SCFAs showed good signals except for some specific samples where SCFAs were detected but not quantified. Figure 9 shows a representative chromatogram of a volunteer before and after consuming dextrin for four weeks. High inter-individual variability was observed, which prevented significant fecal SCFA changes after the administration phase. Besides, the sex of volunteers (all males), Bristol, and BMI values (values were quite homogeneous) were not identified as possible perturbing covariates involved in the variability of SCFA values. There were no significant correlations between SCFA and clinical parameters. 

## 4. Discussion

Our study demonstrates the capability of the microbiota to regulate intestinal gas homeostasis in response to continuous administration of resistant dextrin soluble fibre. The effects of dextrin on the microbiome were monitored online by measuring intestinal gas production, and the adaptive compositional and metabolic remodeling by an integrated metagenomics and metabolomics approach. The relevance of these results is related to the potential role of intestinal gas in abdominal symptoms.

At initial administration, dextrin increased gas production, something plausible because resistant dextrin is fermented by intestinal microbiota releasing gas. However, by four weeks of administration, gas production reverted back to pre-administration levels, expressing the adaptation of the gut microbiota metabolism towards fermentative pathways with lower gas production. Interestingly, the effect persisted and scaled up after discontinuation of administration. This adaptative effect outlasting the administration phase has been also observed with other prebiotics [40,41]. The effects of dextrin on gas production (initial increase and later decay) were dose-dependent: the tendency observed with the low dose in the main study, became statistically significant with the high dose in the ancillary study. Manual counting of the number anal gas evacuations closely correlates with direct measurements of the volume of gas evacuated [16,17,18,19], and in previous studies the effects of dietary interventions on gas metabolism were equally evidenced with both techniques [2,40]. However, in the present study, the effects of dextrin were clearer counting the number of daytime evacuations over the two-day observation periods, particularly with the high dose, than measuring the volume of gas evacuated during the 4-h postprandial period in the gas production tests, conceivably due to the low dextrin dose. At initial administration, dextrin dose-dependently induced gas-related sensations, particularly flatulence, which reverted in parallel to the decay in gas production during subsequent administration and post-administration phase. Dextrin did not interfere with normal gastrointestinal function (bowel habit, and gastric and gallbladder responses to meal ingestion), but these data do not refute that, as with other fibres, it may have an effect in the case of dysfunction. 

The persistent increase in solid colonic content, which was more prominent in the proximal colon, reflects the effects of dextrin on colonic biomass and microbiota. Concerning the microbial community analysis, the metagenomic profiles of each participant reveal relevant changes at taxonomic and functional levels, that can be attributed to the prebiotic effect of dextrin. The alpha diversity estimators described here, were similar to those obtained in other reports in fecal metagenomes [42]. The concomitant increase of 3-HVA and decrease of 7,8-diaminopelargonic acid observed upon dextrin supplementation, could be related to the metabolism of biotin (vitamin B7), a cofactor for essential enzymes in many processes, such as fatty acid biosynthesis, gluconeogenesis, and amino acid metabolism [43]. 

Primary bile acids, synthetized by the liver, are transformed by the gut microbiota to yield secondary bile acids. Secondary bile acids, particularly deoxycholic acid, accumulate in the bile acid pool of individuals on a “Western diet”. Deoxycholic acid is known to activate several cell-signaling pathways associated with disease phenotypes, and increased deoxycholic acid levels in feces, serum, and bile have been reported in patients with colonic cancer and with cholesterol gallstones [44]. Hence, the significant decrease in deoxycholic acid after dextrin administration observed in the present study may have beneficial implications. 

Regarding SCFAs, while some participants showed a consistent increase in the fecal content for almost all SCFAs after dextrin consumption, others showed an opposite behavior. Increase of fecal SCFA concentrations after fibre intervention has been reported in many studies; however, a recent systematic review and meta-analysis described a substantial variability in the reporting of SCFA results, especially in the case of fecal acetate and butyrate [45]. A prebiotic treatment with 16 g/d inulin, similar to the dextrin load in the present study, did not significantly modify fecal SCFA content in obese subjects [46]. However, a higher daily dose of inulin (24 g/d) was reported to increase serum acetate, but not butyrate or propionate in overweight-obese individuals [47]. Our results also agree with [48], who found time- and sex-dependent differences in fecal SCFAs content in 20 healthy volunteers that consumed a soluble fibre (a partially hydrolyzed guar gum, 15 g/d for three weeks, followed by a washout period for three weeks) [48]. These authors observed that men experienced more significant and earlier SCFAs changes during fibre consumption. Remarkably, no SCFAs changes were observed after three weeks of intervention (i.e., acetate increased only after one week, and butyrate and propionate after one and two weeks). These data might explain our results and indicate an adaptation and(or) dynamic flow of SCFAs homeostasis, resulting in better use of SCFAs as an energy source and giving rise to other simpler metabolites.

With regard to microbial community analysis, metagenomic profiles of each participant reveal relevant changes at taxonomic and functional levels that can be attributed to the prebiotic effect in the intervention. Microbial species modulated by resistant dextrin, including *Anaerostipes, Eubacterium,* and *Ruminococcus* species, showed higher abundances in the microbiota of participants showing more favorable values of clinical symptoms. It should be noted that these species comprise some of the major SCFAs producers in human gut. It has been suggested that individuals suffering from disorders involving frequent gastrointestinal symptoms may also show a dysbiosis within the microbiota characterized by reduced abundances of some of these genera [49]. Similarly, the contributions of the *Eubacterium* genus to gut health have been widely studied [50,51].

On the other hand, SCFAs such as acetate, propionate, and butyrate produced by gut microbiota exert numerous benefits on human health. These positive effects include anti-inflammatory activity. In addition, it has been reported that butyrate mediates the differentiation of colonic regulatory T cells while propionate promotes the generation of peripheral regulatory T cells [52]. This biological activity is strongly influenced by the ingestion of complex dietary carbohydrates. In this sense, resistant dextrin promoted the growth of *Anaerostipes*, *Eubacterium*, *Parabacteroides, Roseburia*, and *Ruminococcus* species, which are key bacteria to generate short-chain fatty acids, such as acetate, propionate, or butyrate, according to previous studies [50,52,53,54]; and confirmed the results of a previous study showing the increase of the (Para) bacteroides group by dextrin [55]. 

## 5. Limitations

We wish to acknowledge several limitations of the study. First, this pilot, proof-of-concept study was designed to demonstrate time-dependent variations of microbiota metabolism in response to dextrin administration, by comparing the responses at different time points; no placebo-control was included, but the high dextrin dose tested in the ancillary study provided a dose-control, showing differences with the low dose tested in the main study, even with simultaneous consumption of fiber from other sources. For this exploratory study, a relatively small sample size was included, and given the gender-differences in the responses to food ingestion [56], only men were included for the sake of homogeneity. The sample size was calculated based on the primary outcome, and was insufficient regarding some secondary or exploratory outcomes, which exhibited a high interindividual variability, such as fecal SCFAs content. The results of this pilot study justify and warrant larger, placebo-controlled studies including both genders. Previous studies [40], as well as the ancillary observation, indicate that the adaptation of the microbiota to dietary interventions is fast, but it would also be interesting to investigate the effects of consuming dextrin over an extended period of time, particularly in patients with digestive symptoms.

## 6. Conclusions and Inference

Administration of a resistant dextrin soluble fibre, induced an adaptation of intestinal microbiota and intestinal gas homeostasis, that outlasts the administration phase. Targeted and untargeted metabolomic analyses suggested that consumption of dextrin could enhance short-chain fatty acid production, supported by the increase of some SCFA-producing species. Our results also suggested a slight effect on bile acids and biotin metabolism after dextrin administration. Metagenomic analysis of fecal microbiota revealed the modulatory effect of resistant dextrin on several SCFA-producing bacteria. In this sense, anti-inflammatory butyrate-producing species, such as *Eubacterium eligens*, might be involved in the potential dextrin-induced benefits. Similarly, resistant dextrin modulated a broad range of bacterial metabolic functions involving ribonucleotide, amino acid, and carbohydrate metabolism. 

The adaptation of microbiota metabolism and composition after the four-week dextrin consumption was associated with a shift towards fermentative pathways with reduced gas production. Based on our data in healthy subjects, we cannot ascertain whether this adaptation may have beneficial effects in patients with functional gastrointestinal disorders, characterized by intestinal hypersensitivity and impaired tolerance of intestinal gas. However, other prebiotics, that induce a similar adaptation, have been shown, after a transient worsening at initial administration, to later produce a significant improvement of gas-related symptoms from pre-treatment, persisting after treatment discontinuation [41].

## Figures and Tables

**Table 1:** Microbial taxa modulated by dextrin administration. Mean abundances and standard deviations (SD) of taxonomic clades are compared at basal level, initial, late and post-intervention periods.

		Basal		Initial Intervention		Late Intervention		Post-Intervention	
Level	Taxa	Mean	SD	Mean	SD	Mean	SD	Mean	SD
*Taxonomic clades showing highest abundances during initial intervention period*									
Class	Firmicutes unclassified	1.11	1.20	**2.67**	5.78	2.07	2.97	1.57	1.73
Strain	*Oscillibacter sp.* CAG 241	0.45	0.55	**0.95**	1.73	0.78	1.94	0.61	0.91
*Taxonomic clades showing highest abundances during late intervention period*									
Family	*Tannerellaceae*	2.10	1.91	6.86	8.80	**7.66**	10.07	2.55	4.44
Genus	*Parabacteroides*	2.10	1.91	6.86	8.80	**7.66**	10.07	2.55	4.44
Species	*Parabacteroides distasonis*	1.36	1.44	4.74	6.29	**5.79**	8.16	1.88	4.36
*Taxonomic clades showing highest abundances during post-intervention period*									
Phylum	Firmicutes	39.15	13.15	35.28	12.68	37.83	16.66	**45.63**	18.63
Class	Clostridia	33.13	11.76	28.20	10.91	31.65	14.61	**39.75**	17.17
Order	Clostridiales	33.13	11.76	28.20	10.91	31.65	14.61	**39.75**	17.17
Order	Eggerthellales	0.31	0.28	0.34	0.30	0.41	0.54	**0.44**	0.56
Family	*Eggerthellaceae*	0.31	0.28	0.34	0.30	0.41	0.54	**0.44**	0.56
Family	*Eubacteriaceae*	3.42	2.57	3.05	2.75	2.63	1.89	**4.60**	4.29
Family	*Ruminococcaceae*	11.30	3.58	10.52	4.59	10.79	5.27	**13.52**	7.40
Genus	*Anaerostipes*	0.36	0.51	0.27	0.39	0.46	0.94	**0.62**	0.98
Genus	*Eubacterium*	3.42	2.57	3.05	2.75	2.63	1.89	**4.60**	4.29
Genus	*Lachnospira*	0.49	0.66	0.50	0.69	0.90	1.78	**1.23**	2.32
Genus	*Roseburia*	3.29	3.46	2.46	2.14	3.31	4.03	**4.89**	6.67
Genus	*Ruminococcaceae* unclassified	1.25	3.40	1.73	3.49	1.27	2.62	**2.34**	4.85
Genus	*Ruminococcus*	2.96	3.02	3.15	3.58	3.26	2.63	**4.01**	4.43
Species	*Anaerostipes hadrus*	0.36	0.51	0.27	0.39	0.46	0.94	**0.62**	0.98
Species	*Bifidobacterium longum*	0.60	1.05	0.51	0.74	0.61	1.34	**0.69**	1.62
Species	*Blautia obeum*	0.19	0.21	0.11	0.06	0.17	0.12	**0.26**	0.25
Species	*Dorea longicatena*	0.82	1.08	0.49	0.47	0.48	0.29	**0.94**	1.44
Species	*Eubacterium eligens*	1.26	1.33	1.03	1.39	0.97	0.79	**2.39**	2.47
Species	*Lachnospira pectinoschiza*	0.49	0.66	0.50	0.69	0.90	1.78	**1.23**	2.32
Species	*Roseburia faecis*	0.93	1.19	1.02	1.31	1.91	3.67	**3.14**	5.24
Species	*Roseburia inulinivorans*	0.98	1.07	0.60	0.68	0.69	0.73	**1.12**	2.01
Species	*Ruminococcus bromii*	1.47	2.08	2.16	2.84	1.95	1.98	**2.63**	3.33

**Table 2:** Number of microbial gene families and metabolic pathways showing the highest abundances during initial, late and post-intervention periods. These gene families and metabolic pathways modulated by dextrin administration are summarized by bacterial species.

Initial Intervention		Late Intervention		Post-Intervention	
Microbial Gene Families Modulated by NUTRIOSE^®^ Administration					
Bacterial Species	n	Bacterial Species	n	Bacterial Species	n
*Bacteroides uniformis*	106	*Parabacteroides distasonis*	1837	*Ruminococcus torques*	622
*Firmicutes bacterium CAG 83*	86	*Eubacterium rectale*	860	*Ruminococcus bromii*	617
*Fusicatenibacter saccharivorans*	65	*Fusicatenibacter saccharivorans*	572	*Roseburia inulinivorans*	261
*Parabacteroides distasonis*	63	*Bacteroides uniformis*	395	*Blautia obeum*	201
*Eubacterium rectale*	57	*Bacteroides uniformis* CAG 3	112	*Roseburia faecis*	198
*Parabacteroides merdae*	53	*Ruminococcus torques*	112	*Eubacterium eligens*	142
*Roseburia hominis*	44	*Roseburia inulinivorans*	90	*Dorea longicatena*	134
*Dorea longicatena*	41	*Roseburia hominis*	73	*Coprococcus comes*	123
*Blautia obeum*	37	*Blautia obeum*	44	*Fusicatenibacter saccharivorans*	114
*Coprococcus comes*	29	*Ruminococcus bromii*	44	*Firmicutes bacterium* CAG 83	111
*Roseburia inulinivorans*	29	*Dorea longicatena*	42	*Eubacterium rectale*	97
*Ruminococcus bromii*	27	*Coprococcus comes*	27	*Eubacterium eligens* CAG 72	77
*Firmicutes bacterium* CAG 110	26	*Parabacteroides merdae*	22	*Eubacterium hallii*	67
*Ruminococcus torques*	22	*Roseburia faecis*	17	*Roseburia hominis*	61
*Eubacterium ramulus*	18	*Eubacterium hallii*	16	*Bacteroides uniformis*	43
*Roseburia intestinalis*	14	*Coprococcus catus*	14	*Anaerostipes hadrus*	41
*Eubacterium hallii*	11	*Firmicutes bacterium* CAG 83	9	*Coprococcus catus*	27
*Coprococcus catus*	9	*Eubacterium eligens*	6	*Eubacterium ramulus*	27
*Eubacterium ventriosum*	6	*Eubacterium ramulus*	5	*Firmicutes bacterium* CAG 110	20
*Anaerostipes hadrus*	5	*Eubacterium siraeum*	5	*Eubacterium siraeum*	16
*Bacteroides uniformis* CAG 3	5	*Anaerostipes hadrus*	3	*Eubacterium ventriosum*	15
*Bifidobacterium longum*	5	*Bifidobacterium longum*	3	*Parabacteroides distasonis*	13
*Eubacterium siraeum*	4	*Eubacterium eligens* CAG 72	2	*Roseburia intestinalis*	12
*Roseburia faecis*	2	*Eubacterium ventriosum*	2	*Bifidobacterium longum*	5
*Eubacterium eligens*	1	*Ruminococcus bicirculans*	2	*Roseburia inulinivorans* CAG 15	4
**Total**	**765**	*Coprococcus comes* CAG 19	1	*Ruminococcus bicirculans*	4
		**Total**	**4315**	*Dorea longicatena* CAG 42	3
				*Lachnospira pectinoschiza*	3
				*Coprococcus comes* CAG 19	2
				*Eubacterium hallii* CAG 12	1
				**Total**	**3061**
**Microbial metabolic pathways modulated by NUTRIOSE^®^ administration**					
*Roseburia hominis*	35	*Parabacteroides distasonis*	65	*Roseburia faecis*	52
Firmicutes bacterium CAG 110	1	*Bacteroides uniformis*	58	*Blautia obeum*	50
*Parabacteroides merdae*	1	*Bacteroides uniformis* CAG 3	51	*Eubacterium eligens* CAG 72	39
**Total**	**37**	*Fusicatenibacter saccharivorans*	48	*Anaerostipes hadrus*	36
		*Eubacterium rectale*	41	*Eubacterium eligens*	23
		*Firmicutes bacterium* CAG 83	3	*Ruminococcus bromii*	21
		*Bifidobacterium longum*	1	*Ruminococcus torques*	11
		*Bifidobacterium longum* CAG 69	1	*Dorea longicatena*	6
		*Ruminococcus bicirculans*	1	*Dorea longicatena* CAG 42	6
		**Total**	**269**	*Roseburia inulinivorans*	5
				*Coprococcus comes*	3
				*Roseburia inulinivorans* CAG 15	2
				*Coprococcus catus*	1
				*Eubacterium hallii*	1
				*Eubacterium siraeum*	1
				*Lachnospira pectinoschiza*	1
				**Total**	**258**

## Data Availability

Raw sequences data deposited in the Sequence Read Archive (SRA) of the NCBI (https://www.ncbi.nlm.nih.gov/sra, accessed on 25 October 2022).

## Supplementary Materials

The following supporting information can be downloaded at: https://www.mdpi.com/article/10.3390/nu14214611/s1: Effects induced by a pleasant meal. Analytical methods. Supplementary Figure S1: Bowel habit with 14 g/d dextrin. Supplementary Figure S2: Bowel habit with 42 g/d dextrin. Supplemental Figure S3: Clustering of analysis of taxonomic profiles (A), gene families (B) and metabolic pathways (C). Supplementary Figure S4: Principal coordinates analysis of taxonomic profiles (A), gene families (B) and metabolic pathways (C). Supplementary Figure S5: Venn diagrams showing number of urine metabolites.
