# Supplementary material for: Effect of Resistant Dextrin on Intestinal Gas Homeostasis and Microbiota
Source: Nutrients. 2022 Nov 2;14(21):4611. doi: 10.3390/nu14214611 (PMC9654059; doi:10.3390/nu14214611)
Supplement: Supplementary file 1 [file nutrients-14-04611-s001.zip › nutrients-1978430-supplementary.pdf]

SUPPLEMENTARY MATERIALS

Effects induced by a pleasant meal

Methods

The third day of each evaluation period (at the end of the pre-administration phase, initial administration phase and late administration phase) the biological response to a comfort meal (sensations and digestive function) was tested (Figure S1). The *comfort meal* consisted of a warm sandwich (58 g bread with 12 g butter, 38 g ham and 38 g cheese) and 200 mL orange juice (300 mL total volume, total caloric content of 425 Kcal; 17 g lipids, 47 g carbohydrates, 18 g proteins).

Digestive sensations were measured using graded scales, as follows. Two 10 cm scales graded from 0 (not at all) to 10 (very much) were used to measure abdominal fullness and discomfort/pain. Four additional 10 cm scales graded from +5 to -5 were used to measure homeostatic and hedonic sensations: hunger/satiety (from extremely hungry to completely sated), desire of eating a food of choice (from eagerly to impossible), digestive well-being (from extremely pleasant sensation/satisfaction to extremely unpleasant sensation/dissatisfaction) and mood (from very positive to very negative). Subjects received standard instructions on how to fill out the scales. The scales were scored before ingestion and at 0 min, 30 min and 60 min after ingestion.

Antral and gallbladder emptying were measured by ultasonography. In brief, ultrasound images of the gastric antrum and the gallbladder were obtained using a Chison ultrasound scanner (ECO1; Chison, Jiangsu, China) with an abdominal 3.5-Hz probe (C3A; Chison, Jiangsu, China); images were obtained with the subjects seated and leaning slightly backwards in a chair. Gastric images

between antral contractions were obtained in triplicate before and at 5 minutes, 30 minutes, and 60 minutes after the meal; using the superior mesenteric vein and the aorta as landmarks, the outer profile and the cross-sectional area of the antrum were measured using the built-in calliper and measurement tool. Images of the gallbladder were obtained before and 60 minutes after the meal to measure the longitudinal axis.

Results

In the pre-administration phase, ingestion of the comfort meal induced satiety ($p<0.001$) and reduced the desire of choice eating ($p<0.001$), without significant fullness sensation ($p=0.135$) or discomfort ($p=0.439$), and enhanced digestive well-being ($p=0.021$) and mood ($p=0.022$). These sensations declined during the postprandial period. Ultrasonography showed: a) an increase in intragastric content immediately after meal ingestion ($p<0.001$ vs fasting) followed by a gradual decrease during the postprandial period (by 30$\pm$13% at 60 min; p= 0.003); b) a contraction of the gall bladder measured as a significant reduction in the longitudinal axis at 60 min after ingestion as compared to before ingestion; $p=0.002$). Administration of soluble resistant dextrin (NUTRIOSE® 14 g/d) did not induce consistent in changes in the sensations induced by the comfort meal, and did not disturb the normal gastric and gall bladder emptying rates either at the initial or at the late administration phase.

Analytical methods

Urine UPLC-ESI-QTOF-MS analysis

Urine samples (with and without enzymatic treatment) were injected into a reverse-phase column, a Poroshell 120 EC-C18 column (3 x 100 mm, 2.7 µm)

(Agilent), operating at 30 ºC and a flow rate of 0.5 mL/min. The mobile phases used were acidified water (0.1% formic acid) (Phase A) and acidified ACN (0.1% formic acid) (Phase B). Compounds were separated using the following gradient conditions: 0–3 min, 5–18% phase-B; 3–10 min, 18–50% phase-B; 10–13 min, 50–90% phase-B. Finally, phase B content was returned to the initial conditions (5%) for 1 min, and the column re-equilibrated for two more minutes. Data were acquired using the Mass Hunter Workstation software (version B.08.00, Service Pack 1, Agilent Technologies). The system was operated using both negative and positive ion polarity. The operating conditions were as follows: gas temperature of 280 ºC, drying nitrogen gas of 9 L/min, nebulizer pressure of 45 psi, sheath gas temperature of 400 ºC, sheath gas flow of 12 L/min, a capillary voltage of 3500 V, nozzle voltage of 500 V, fragmentor voltage of 100 V, skimmer of 65 V and octopole radiofrequency voltage of 750 V. TOF spectra acquisition rate/time was 1.5 spectra/s and 666.7 ms/spectrum respectively, and transients/spectrum were 5484. The mass range was between 50 and 1100 *m/z*. At the beginning of the batch, the instrument was calibrated to assure mass accuracy during the MS analysis using a mixture of reference compounds (Tuning Mix). Continuous internal calibration was performed during analyses using signals *m/z* 112.9855 and *m/z* 1033.9881 in negative polarity and *m/z* 121.0509 and *m/z* 922.0098 in positive polarity. Auto recalibration reference-mass parameters were a detection window of 100 ppm and a minimum height of 1000 counts. MS/MS conditions were collision energy 20 eV and acquisition time 100 ms/spectrum. Data were processed using the Mass Hunter Qualitative Analysis software (version B.08.00, Service Pack 1, Agilent Technologies). As there were no specific target analytes (untargeted analysis), generic settings were applied to

obtain as many compounds as possible. QTOF-MS offers high selectivity, sensitivity, resolution, and high mass accuracy, providing a powerful tool for complex samples' metabolic profiling. All samples were injected in the same batch, and the order of sample injection was randomized to avoid sample bias. A mixture with one replicate of each group of samples was used as 'quality control' (QC) and was injected at the beginning and the end of the batch. Besides, MeOH injections were included every three samples as a blank run to avoid the carry-over effect.

Feature extraction was carried out on Agilent Profinder B.06.00, a stand-alone feature extraction program for LC-MS based profiling analyses. To find compounds by Molecular Features (MFs) was carried out using a pre-filter to take peaks with a height greater or equal to 10,000 counts, allowing only –H and +HCOO as negative ions species and +H as positive ions. Unidentified compounds were aligned across the different samples based on their retention times' tolerance and the mass spectral similarity. The MassHunter MSC (Molecular Structure Correlator) program was used to correlate accurate mass MS/MS fragment ions for a compound of interest with one or more proposed molecular structures for that compound.

Bile acids were analyzed in urine using the same LC system and phase-reverse column, but with some protocol modifications. The mobile phases used were acidified water (0.1% formic acid) (Phase A) and acidified ACN (0.1% formic acid) (Phase B). Compounds were separated using the following gradient conditions: 0–4 min, 2–18% phase-B; 4–8 min, 18–36% phase-B; 8–11 min, 36–55% phase-B, 11–14 min, 55–90% phase-B. Finally, phase B content was returned to the initial conditions (2%) for 2 min, and the column re-equilibrated for

two more minutes.

Urine and fecal metabolomics analysis by GC-MS

An HP5-MS (30 m x 0.25 mm ID and film thickness of 0.25 µm, Agilent) phase capillary column was used with helium as a carrier gas at a constant rate of 1 mL/min. The temperatures of the injector and MS source were maintained at 200 and 250 °C, respectively. A glass liner with a glass wool plug at the lower end of the liner was used to avoid the contamination of the GC column with nonvolatile urine material. Every five samples injected, a blank sample with hexane was inserted to check for memory effects. The column temperature program consisted of injection at 60 °C (hold time 1 min), which was raised at 7 °C/min to 180 °C, at 3 °C/min to 200 °C (hold time 1 min), and finally at 10 °C/min to 230 °C (hold time 10 min). Ferulic, vanillic and caffeic acids (20 µg) were used as internal standards. The samples were analyzed in split mode (split ratio: 1/10). The MS was operated in the electron impact mode with an ionization energy of 70 eV. The mass scan range was set from 50 to 800 Da at 2.05 scan/s.

Data pretreatment for untargeted metabolomics

Batch Feature Finding: MassHunter Profinder

Feature extraction was carried out on Agilent Profinder B.06.00 (Agilent Technologies, Waldbronn, Germany), a stand-alone feature extraction program for LC/MS-based profiling analyses. Profinder was optimized to extract features from large data sets and provide an intuitive user interface to inspect and review each feature across the files associated with the data set. Extracted ion chromatograms and mass spectral data related to each feature were revised and compared simultaneously, and scored by the software.

Once the data files were selected, the recursive feature extraction workflow algorithm was selected, and then, the method for the feature extraction algorithm was edited and reviewed. To find compounds by Molecular Features (MFs) was carried out using a pre-filter to take peaks with a height greater or equal to 10000 counts, allowing only –H and +HCOO as negative ions species and +H as positive ions, a peak spacing tolerance of 0.0025 *m/z* plus 7.0 ppm, and charge states limited to a maximum of 2. The file obtained with this pre-processing was created for each sample as a CEF file. The final files were exported into the Mass Profiler Professional (MPP) software package (revision B.14.09.01, Agilent Technologies, Santa Clara, CA, USA) for statistical analysis.

A new experiment typed as unidentified was created in MPP, and significance testing and fold change were done. The data source used for the investigation was MassHunter Qual, and the organism was *Homo Sapiens*. In the second step, data were imported from files. The next steps were sample reordering and experiment grouping. After data were imported, several filtering options were applied, such as a minimum absolute abundance of 5000 counts, minimum mass filtering of 70 and maximum mass filtering of 1200, numbers of ions required greater or equal to 2, and the option to all charge states forbidden.

Alignment, Normalization and Baseline Transformation.

The next step was the alignment of parameters, where unidentified compounds were aligned across the different samples based on their retention times' tolerance and the mass spectral similarity. Compounds from different samples with the same *m/z* (mass tolerance window: 5 ppm, 2 mDa) and retention time (tolerance window: 0.15 min) were considered the same. Next, the baselining pre-processing options were selected. The goal of normalization is to

limit systematic non-biological variation to reveal real biological variation. Normalization steps were performed ‘within samples’ whereas baselining was ‘per entity’. Using baseline transformation improved both visualization of abundance pattern similarities and grouping of masses with similar abundance patterns in clustering analysis. Data were transformed to the log 2 scale and centred to the median across samples set to lower the relatively significant differences in the respective MFs abundance. All compounds were treated equally regardless of their abundance.

Quality Control on Samples and Entities

Quality control can be performed at both the sample and mass levels to eliminate low-quality samples and unreliable measurements. Carrying over low-quality samples and masses could limit statistically significant findings or informative clustering results. ‘Filter on frequency, on flags and by abundance’ criteria were applied to reduce the dimensionality of the data and select the most representative compounds, which are rarely detected (therefore not very reliable) and filter out entities with non-reproducible measurements within a condition. Only the MFs present in at least 80% of replicates in at least one condition were considered. This entity filtering allowed creating a higher quality data set so that the following multivariate analysis should be more significant.

Compound identification: Molecular structure correlator.

The MassHunter MSC (Molecular Structure Correlator) program was used. This software accurately correlated mass MS/MS fragment ions for a compound of interest with one or more proposed molecular structures. Then, each selected formula was used to retrieve one or multiple possible structures

from several on-line database and libraries, including the MassHunter compound database (PCDL), METLIN/ChemSpider/KEGG/Lipid Maps, etc., and score how well each candidate structure correlated with the MS/MS spectrum.

The validation of tentatively identified compounds by the MassHunter program was carried out (whenever possible) by chromatographic comparison with authentic standards. When authentic standards were not available, indirect validation was tried according to the fragmentation by MS/MS and metabolites' elution order.

Supplementary figures

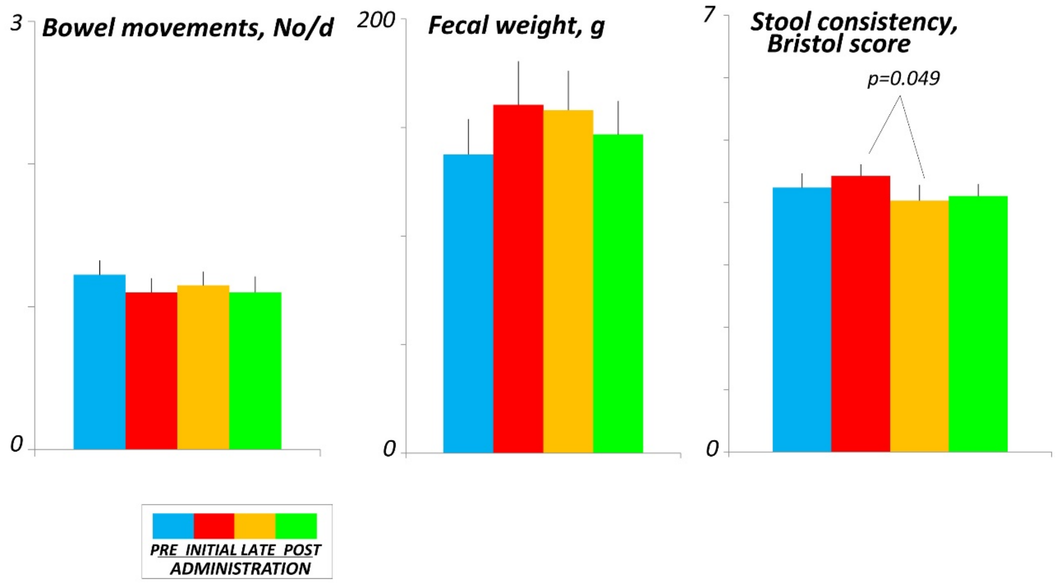

Supplementary Figure S1. Bowel habit with 14 g/d dextrin. Data (number of bowel movements, fecal weight and stool consistency score) are average of 2 consecutive days of the evaluation periods in main study.

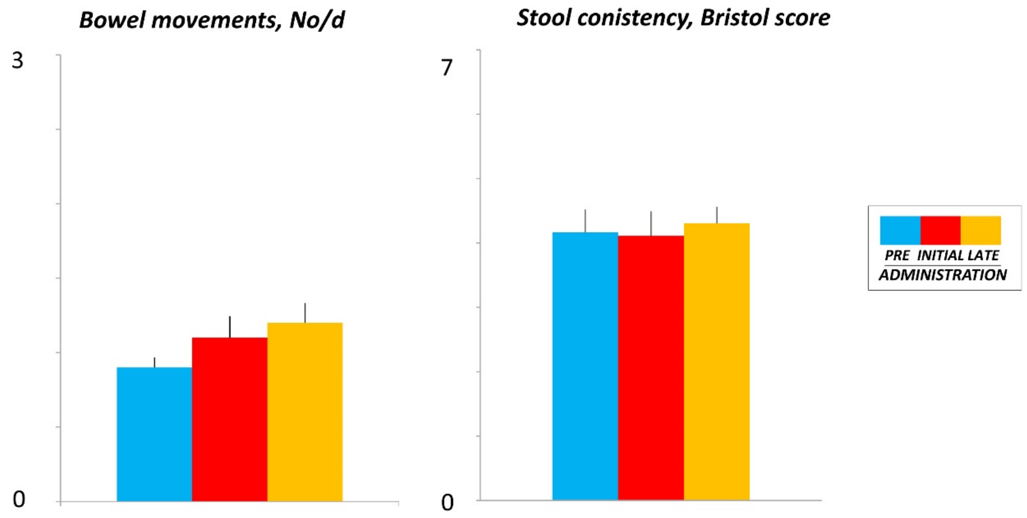

Supplementary Figure S2. Bowel habit with 42 g/d dextrin. Data (number of bowel movements and stool consistency score) are average of 2 consecutive days of the evaluation periods in ancillary study with triple dose of dextrin.

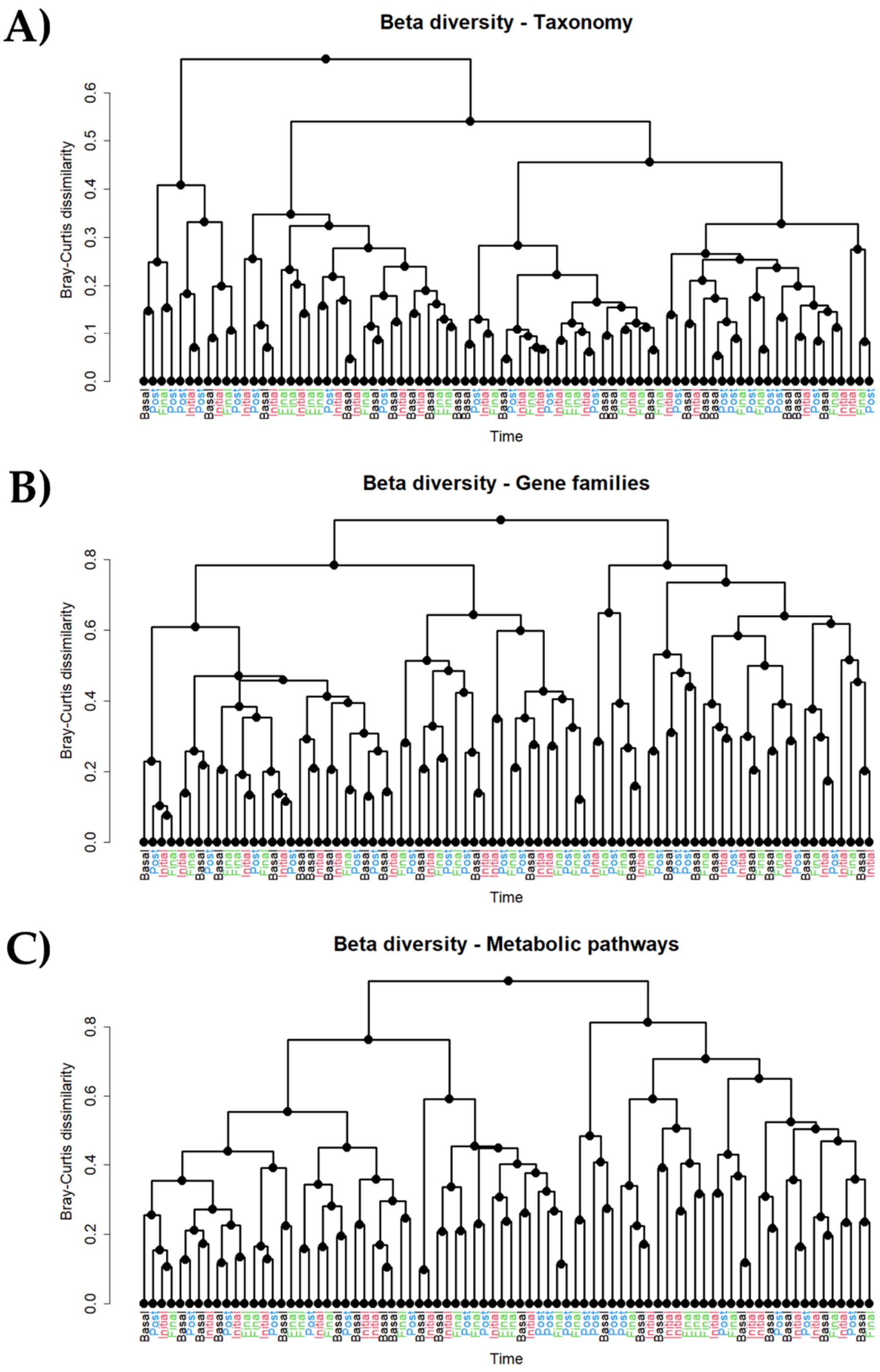

Supplementary Figure S3. Clustering of analysis of taxonomic profiles (**A**), gene families (**B**) and metabolic pathways (**C**) found in the microbiota of participants at different intervention periods: basal, initial intervention (initial), late intervention (final) and post-intervention (post). Bray-Curtis dissimilarity method was selected for the calculation. As it can be seen, few samples corresponding to the same intervention period were clustered together. The rest of samples were clustered together with metagenomes from the same participant taken at different periods, highlighting the role of interindividual variability.

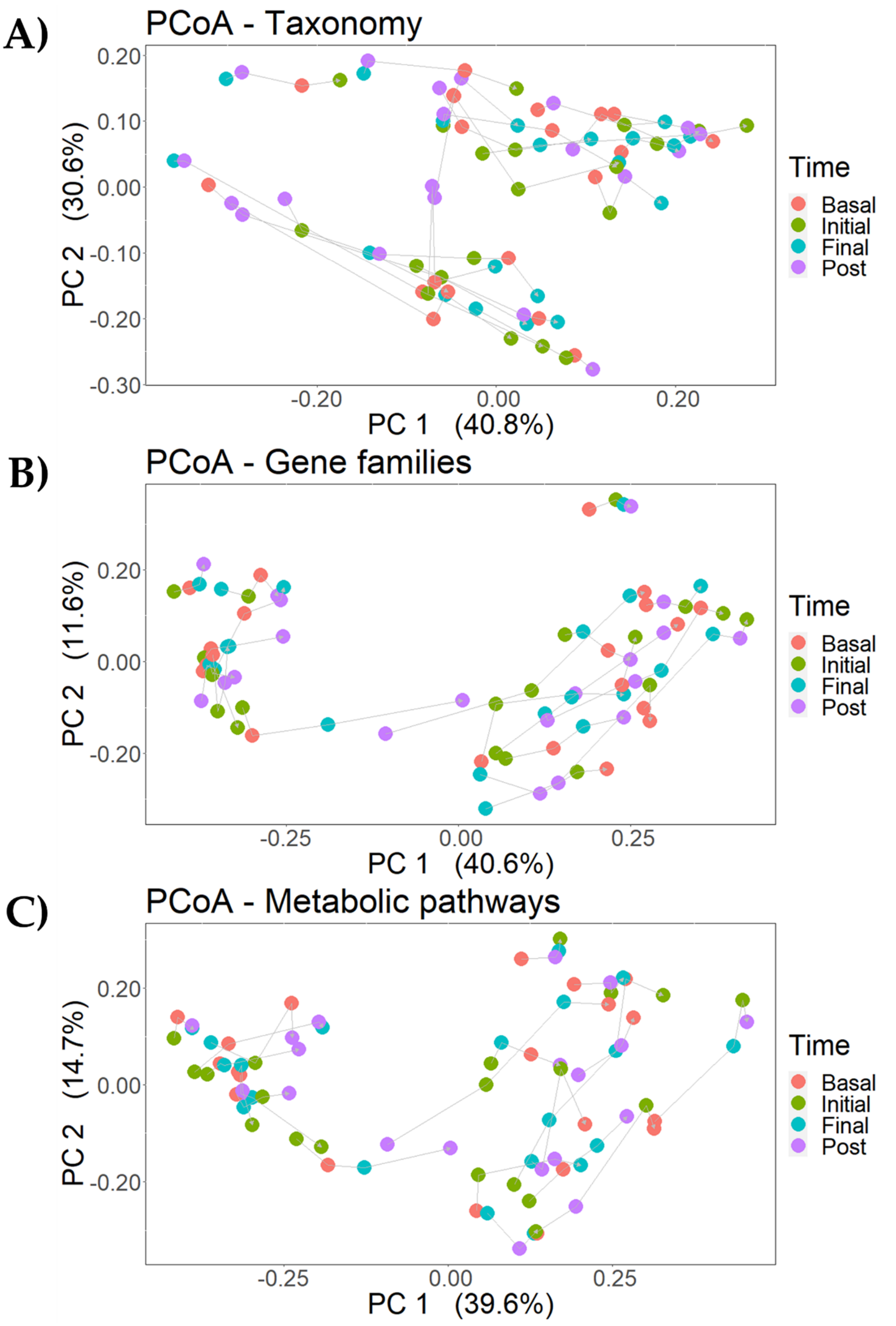

Supplementary Figure S4. Principal coordinates analysis (**PCoA)** of taxonomic profiles (**A**), gene families (**B**) and metabolic pathways (**C**) found in the microbiota of participants at different intervention periods: basal, initial intervention (initial), late intervention (final) and post-intervention (post). **PC:** principal coordinate. The percentage of variance explained by each PC is indicated in the axis.

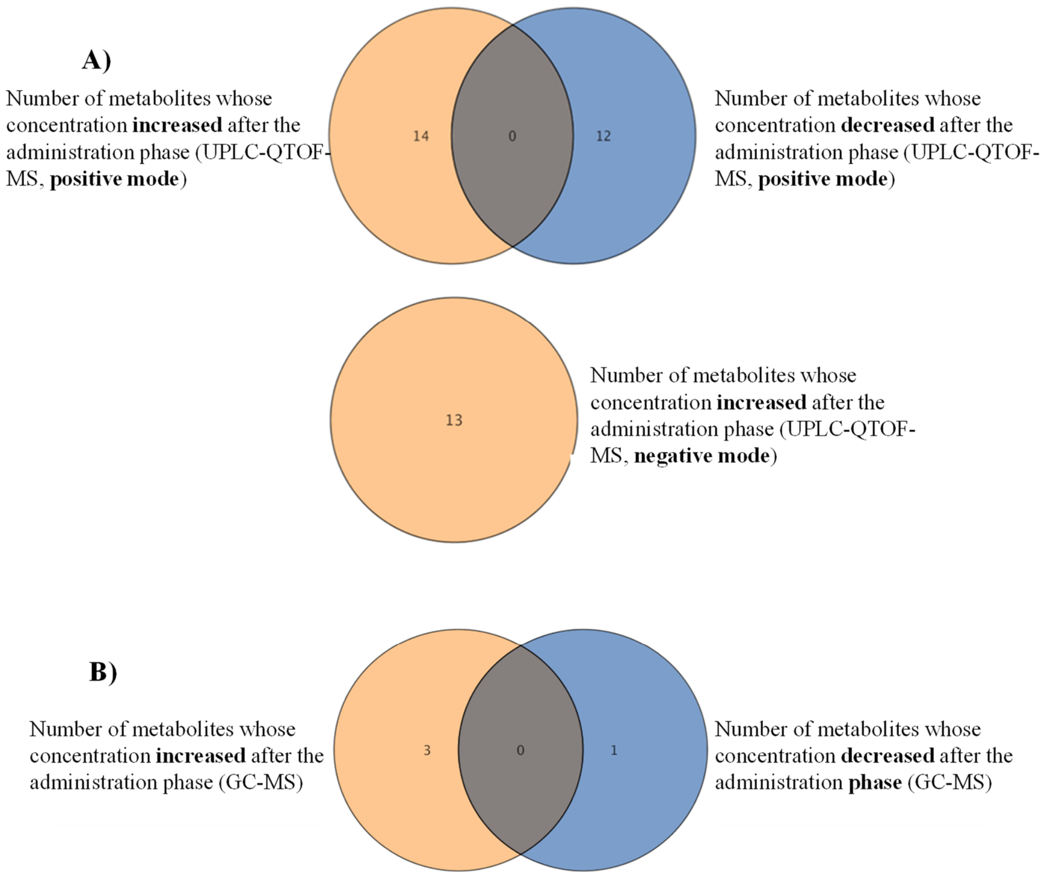

Supplementary Figure S5. Venn diagrams showing number of urine metabolites. **A**) Metabolites, measured by UPLC-QTOF-MS, with significantly different concentration after the administration phase (in both positive and negative ionization modes). **B**) Metabolites, measured by GC-MS, with significantly different concentration after the administration phase.
